# Finerenone effects on biomarkers: an analysis from the FIGARO-DKD trial

**DOI:** 10.1093/eurheartj/ehaf316

**Published:** 2025-05-26

**Authors:** Mario Berger, Aidan MacNamara, João Pedro Ferreira, Peter Kolkhof, Sebastian Voss, Adam Skubala, Andrea Scalise, Laura Goea, Richard Nkulikiyinka, Bertram Pitt, Joachim Hanno Ix, Peter Rossing, Richard John Mark Coward, Faiez Zannad, Hiddo J L Heerspink

**Affiliations:** Bayer AG, Pharmaceuticals, R&D, Wuppertal/Berlin, Germany; Bayer AG, Pharmaceuticals, R&D, Wuppertal/Berlin, Germany; Department of Surgery and Physiology, Faculty of Medicine of the University of Porto, Cardiovascular Research and Development Center (UnIC@RISE), Porto, Portugal; Inserm, Université de Lorraine, Centre d'Investigations Cliniques, Plurithématique 14-33, and Inserm U1116, CHRU Nancy, F-CRIN INI-CRCT (Cardiovascular and Renal Clinical Trialists), Nancy, France; Bayer AG, Pharmaceuticals, R&D, Wuppertal/Berlin, Germany; Chrestos GmbH, Biomarker Statistics and Data Science Department, Essen, Germany; Bayer AG, Pharmaceuticals, R&D, Wuppertal/Berlin, Germany; Bayer Hispania, S.L., Clinical Development, Barcelona, Spain; Bayer AG, Pharmaceuticals, R&D, Wuppertal/Berlin, Germany; Bayer AG, Pharmaceuticals, R&D, Wuppertal/Berlin, Germany; Department of Medicine, University of Michigan School of Medicine, Ann Arbor, MI, USA; Department of Medicine, University of California San Diego and Veterans Affairs San Diego Healthcare System, San Diego, CA, USA; Steno Diabetes Center Copenhagen, Herlev, and University of Copenhagen, Copenhagen, Denmark; Bristol Renal, University of Bristol, Bristol, UK; Inserm, Université de Lorraine, Centre d'Investigations Cliniques, Plurithématique 14-33, and Inserm U1116, CHRU Nancy, F-CRIN INI-CRCT (Cardiovascular and Renal Clinical Trialists), Nancy, France; Department of Clinical Pharmacy and Pharmacology, University Medical Center Groningen, Groningen, The Netherlands

**Keywords:** Extracellular matrix remodelling, Fibrosis, Finerenone, MRA, Neuropeptide Y, Proteomics

## Introduction

Finerenone is a non-steroidal mineralocorticoid receptor antagonist (MRA) selective for the MR.^1^ In three large outcome trials, finerenone improved cardiovascular (CV) and kidney outcomes in patients with chronic kidney disease (CKD) and Type 2 diabetes (T2D), and reduced the risk for the composite endpoint of total heart failure events and CV death in heart failure patients with ejection fraction ≥40.^2–4^ In animal models of cardiorenal disease, finerenone treatment ameliorated cardiac and renal hypertrophy, reduced sodium retention and proteinuria. Along with histological findings of structural improvement in heart and kidneys, several inflammation and fibrosis-associated biomarkers were reduced by finerenone.^1^ Less is known about the proteomic profile of finerenone in humans. Hence, this study describes the longitudinal effects of finerenone on the plasma proteome in patients with CKD and T2D.

## Methods

This is a *post hoc* analysis of the biomarker sub-study to the Phase III trial, FIGARO-DKD.^3^ It included 929 subjects from 115 clinical sites in 21 countries; site selection was based on above-average recruitment. 2941 biomarkers were measured in a total of 4193 samples on Olink Explore3072. All participants had been on treatment with either placebo or finerenone for ≥24 months and samples from the *first* post-randomization visit (month 4[M4]), up to month 48 after treatment initiation were analysed. Baseline samples were not available.

Finerenone effects were assessed using linear mixed models for repeated measurements including treatment, visit, their interaction, and sex as fixed effects. Biomarkers were considered responsive to finerenone (vs placebo) if between-group differences in least squares means were statistically significant on at least one sampling timepoint (4–48 months) after adjusting for multiple testing [false discovery rate (FDR) *q* ≤ 0.01].

Finerenone-responsive biomarkers were used for pathway enrichment with the R package bc3net.^5^ Briefly, known biological pathways were queried to test for enriched pathways in the input set of biomarkers while adjusting for background to account for the bias in protein selection within Olink’s platform.

## Results

The median age of study participants was 64 years (interquartile range 59–70) and 75% were men. At baseline, the median estimated glomerular filtration rate (eGFR) was 65.3 mL/min/1.73 m^2^ (interquartile range, 49.4–81.3), >97% of subjects were albuminuric (urinary albumin-to-creatine ratio ≥ 30 mg/g), and overall comparable to the parent trial.^3^ 17.3% of patients were hospitalized for cardiovascular diseases (CVD) and 34.6% had a history of CVD prior to randomization. All participants had CKD and T2D, and were on renin-angiotensin-aldosterone-system (RAAS) inhibitors.^3^ Of 929 patients, 474 (51%) received finerenone and 455 (49%) placebo.

**Figure 1:**
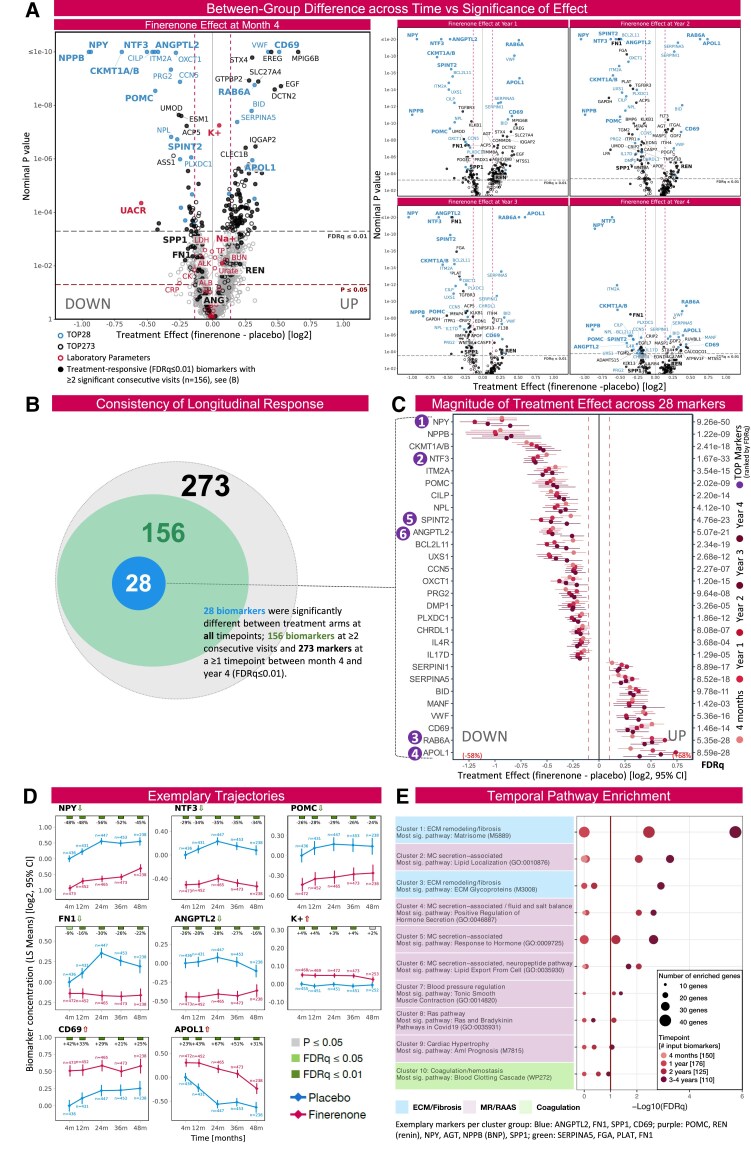
Finerenone elicits a stable longitudinal biomarker response and appears to modulate remodelling and RAAS-associated pathways. (*A*) Volcano plots illustrating the treatment effect of finerenone on biomarkers and routine laboratory parameters across time. *X*-axis: Difference in treatment effect estimates between finerenone and placebo group from linear mixed models (on log2 scale). *Y*-axis: nominal *P*-value (−log10). TOP28 markers (i.e. biomarkers which demonstrated a statistically significant treatment response at the level of 1% after adjusting for multiple testing (finerenone vs placebo, false discovery rate [FDR] *q* ≤ 0.01) at all timepoints from Month 4 to Year 4) are shown in blue, remaining TOP273 markers (FDR*q* ≤ 0.01 at ≥1 timepoint) in black, Lab parameters in red and all remaining markers of the Olink panel in grey. Filled symbols (•) indicate biomarkers with a significant between-group difference (FDR*q* ≤ 0.01) at ≥2 consecutive timepoints. Biomarker labelling threshold: *P* ≤ 10e-6. Markers in bold print are mentioned in the manuscript text. (*B*) Venn diagram showing the number of significantly modulated biomarkers at ≥1 (TOP273), ≥2 or 5 timepoints (TOP28; finerenone vs placebo, FDR*q* ≤ 0.01). (*C*) Forest plot showing the TOP28 biomarkers ranked by effect size vs the treatment effect difference from the linear mixed models as in (*A*) for each timepoint. Lowest FDR*q* (Month 4 to Year 4) is given for each marker; significance ranks 1–6 are highlighted in purple. (*D*) Time trajectories of biomarker concentrations given as Least Squares Means (adjusted, log2-scaled NPX units, zero-centered to placebo at Month 4) by treatment arm: finerenone (magenta), placebo (blue). Relative difference (%) between finerenone and placebo is shown at the top along with *P* and FDR*q* value (based on the between-group difference from linear mixed models). N (next to marker): number of measurements (subjects) per timepoint. (*E*) Bubble plot of enriched pathway clusters: Sets of treatment-responsive markers at each given timepoint were used for temporal pathway enrichment and the resulting pathways (*P* < .01) were grouped by similarity (i.e. how similar each pathway is in terms of member biomarkers), ranked by FDR*q* value. The top ten pathway families are shown along with the most significant pathway (at any point in time) in each cluster (with pathway ID).^6^ Clusters were manually grouped (blue, purple, and green) and annotated based on review of member pathways. Exemplary biomarker names are given for the three groups: ECM/fibrosis, MR/RAAS, Coagulation. AGT, angiotensinogen; ALB, albumin (serum or plasma); ALK, alkaline phosphatase; ANGPTL2, angiopoietin-related protein 2; APOL1, Apolipoprotein-L1; BUN, blood urea nitrogen; CK, creatine kinase; CRP, C-reactive protein; ECM, extracellular matrix; FDR*q*, false discovery rate *q* value; FGA: Fibrinogen, alpha chain; FN1, fibronectin; K^+^, potassium (serum); LDH, lactate dehydrogenase; LS, Least Squares (Means); MC, mineralocorticoid; Na^+^, sodium (serum); NPPB, B-type natriuretic peptides (BNP); NPX, Normalized Protein eXpression (Olink unit); NPY, neuropeptide Y; NTF3, neurotrophin-3; PLAT, tissue-type plasminogen activator; POMC, pro-adrenocorticotropin; sig., significant; SPP1, osteopontin; TP, total protein (serum); UACR, urinary albumin-to-creatinine ratio. Otherwise gene names are used. Vertical lines (*A*–*C*) ±10% treatment effect threshold (on linear scale)

Renin-angiotensin-aldosterone-system effectors like renin, its substrate angiotensinogen, and the precursor of the key aldosterone stimulator adrenocorticotropin, POMC, were consistently modulated by finerenone, along with subtle changes in electrolytes (K^+^/Na^+^), indicative of MR blockade and feedback regulation (*Figure 1A*). Altogether, 273 biomarkers differed between treatment arms (FDR*q* ≤ 0.01) at ≥1 timepoint, 156 markers at ≥2 consecutive timepoints, and 28 markers at all timepoints up to year 4 (*Figure 1A and B*). *Figure 1C* shows the treatment effect magnitude for the most consistently different 28 markers. Among these, neuropeptide Y (NPY), B-type natriuretic peptides, and U(biquitous)-type mitochondrial creatine kinase (CKMT1A/B) appeared most-downregulated by finerenone, whereas apolipoprotein-L1 (APOL1), the small GTPase Rab-6A (RAB6A) and CD69 were most elevated in finerenone-randomized patients. Overall, biomarkers with most significant between-group difference (FDR*q* < 10^−20^ at any timepoint) were: NPY, neurotrophin-3 (NTF3), RAB6A, APOL1, the protease inhibitor SPINT2, and angiopoietin-related protein 2 (ANGPTL2). Exemplary trajectories are shown in *Figure 1D*.

We then performed pathway enrichment. Extracellular matrix remodelling pathways (blue clusters, *Figure 1E*) were most significantly enriched at ≥2 years after treatment initiation (FDR*q* < 0.01). Drivers of this enrichment included fibronectin and osteopontin; both are well-known fibrosis biomarkers and were consistently lower in the finerenone arm. Other enriched pathways included mineralocorticoid secretion and its haemodynamic actions (purple clusters), re-affirming target engagement, and also coagulation/hemostasis (green).

## Discussion

Finerenone appeared to modulate a multitude of circulating proteins. Pathway enrichment suggested that many of them are involved in haemodynamic control and fibrosis-associated processes, supporting previous literature on antifibrotic and RAAS-modulating properties of MRAs.^1,7^ Interestingly, NPY, a neurohormone with pleiotropic functions and vasoconstrictor properties, was strongly suppressed by finerenone in this population. Elevated plasma NPY has been associated with adverse cardiac remodelling^8,9^ and kidney disease progression.^10^ Mechanistic work demonstrated that knockout or blockade of NPY protects from albuminuric kidney disease.^10^ It has also been suggested that NPY can directly modulate adrenal aldosterone release,^11^ but not thus far that altered aldosterone or MR blockade affect circulating NPY. Downregulation of NTF3 by finerenone may be linked as vascular-derived NTF3 reportedly modulates NPY in sympathetic neurones innervating blood vessels,^12^ thereby likely affecting vascular tone. Other treatment-responsive markers detected included CD69 (T cell activation marker), ANGPTL2 (proinflammatory adipokine), CKMT1A/B (ubiquitous mitochondrial creatine kinase), RAB6 (intracellular transporter) and APOL1 (component of HDL cholesterol). CD69 and ANGPTL2 have previously been studied in the context of immunomodulation in CKD/CVD.^13–15^ CKMT1A/B is central for cellular energy homeostasis by forming phosphocreatine which serves as fuel for tissues with high energy demand.^16^ RAB6 is involved in vesicular traffic in secretory pathways and mediates NPY exocytosis *in vitro*.^17^ Rab GTPases also participate in aldosterone-dependent relocalization of ion channels^18^ suggesting a possible crosstalk between aldosterone/MR and upstream neurohormonal secretion. Apolipoprotein-L1 dysfunction has been heavily implicated in proteinuric CKD progression in African-Americans.^19^ Exploring possible mechanistic roles of these markers and their interplay in cardiorenal disease progression will be of interest.

Although we compared plasma biomarker levels between treatment arms without adjusting for interindividual differences at baseline, randomization into the parent trial is expected to result in balanced phenotypes and biomarker levels at treatment start. Consequently, any differences between treatment groups at later timepoints are likely true differences. Sensitivity analyses adjusting for baseline CV risk factors or investigating effects post-month 4 provided highly comparable results supporting this assumption (data not shown). We acknowledge that the results presented here should be further validated in mechanistic models and prospective randomized trials to address their clinical relevance and how they may impact future clinical practice.

In conclusion, finerenone’s action is not restricted to RAAS and downstream targets of the MR/aldosterone transcription factor complex but extends to upstream neurohormonal, hemostasis, energy metabolism, immune-related and remodelling-associated pathways. Their modulation may contribute to finerenone’s clinical benefits in patients with cardiorenal disease.

## Declarations

### Data Availability

Availability of the data underlying this publication will be determined according to Bayer’s commitment to the EFPIA/PhRMA ‘Principles for responsible clinical trial data sharing’. This pertains to scope, timepoint and process of data access. As such, Bayer commits to sharing upon request from qualified scientific and medical researchers patient-level clinical trial data, study-level clinical trial data, and protocols from clinical trials in patients for medicines and indications approved in the United States (US) and European Union (EU) as necessary for conducting legitimate research. This applies to data on new medicines and indications that have been approved by the EU and US regulatory agencies on or after 01 January 2014. Interested researchers can use www.vivli.org to request access to anonymized patient-level data and supporting documents from clinical studies to conduct further research that can help advance medical science or improve patient care. Information on the Bayer criteria for listing studies and other relevant information is provided in the member section of the portal. Data access will be granted to anonymized patient-level data, protocols and clinical study reports after approval by an independent scientific review panel. Bayer is not involved in the decisions made by the independent review panel. Bayer will take all necessary measures to ensure that patient privacy is safeguarded.

### Pre-registered Clinical Trial Number

The pre-registered clinical trial number is NCT05013008. https://clinicaltrials.gov/study/NCT05013008.
